# Knowledge, attitudes, and practices of healthcare providers in Beijing regarding human immunodeficiency virus and tuberculosis co-infection: A multicenter cross-sectional study

**DOI:** 10.1371/journal.pone.0341132

**Published:** 2026-02-23

**Authors:** Yuwei Liu, Jinghe Liu, Yufei Chang, Xiaoyou Chen

**Affiliations:** 1 Tuberculosis Department, Beijing Chest Hospital, Capital Medical University, Beijing Tuberculosis and Thoracic Tumor Research Institute, Beijing, China; 2 Emergency Department, Beijing Ditan Hospital, Capital Medical University, Beijing, China; 3 Infectious Diseases Department, Beijing Ditan Hospital, Capital Medical University Beijing, Beijing, China; Universitas Indonesia Fakultas Kedokteran, INDONESIA

## Abstract

**Background:**

Human Immunodeficiency Virus (HIV) and tuberculosis (TB) co-infection poses a significant challenge to public health systems due to its complex clinical management and high mortality. This study aimed to reveal the specific mechanisms through which knowledge influences practice in HIV/TB co-infection management among healthcare providers (HCPs) using structural equation modeling (SEM).

**Methods:**

An exploratory cross-sectional study using convenience sampling was conducted from May to June 2025 involving healthcare providers (HCPs) across various medical institutions in Beijing, which included university-affiliated tertiary hospitals, specialized hospitals, and community health centers.

**Results:**

A total of 565 valid questionnaires were collected, with 364 (64.42%) completed by medical doctors. The knowledge, attitude, and practice scores were 18.51 ± 7.75 (possible range: 0–30), 43.64 ± 5.51 (possible range: 10–50), and 30.75 ± 7.44 (possible range: 8–40), respectively. Spearman correlation analysis revealed significant positive correlations between knowledge and attitude (r = 0.500, P < 0.001), between attitude and practice (r = 0.584, P < 0.001) and between knowledge and practice (r = 0.592, P < 0.001). SEM analysis indicated that knowledge had a direct influence on both attitudes (β = 0.458, P = 0.002) and practices (β = 0.491, P = 0.011), while attitudes influenced practices (β = 0.272, P = 0.008). Furthermore, knowledge indirectly affected practices through attitudes (β = 0.124, P = 0.005).

**Conclusion:**

In our sample, respondents demonstrated limited knowledge, generally positive attitudes, and moderately adequate practices regarding HIV and TB co-infection. These preliminary findings suggest that targeted educational interventions designed to enhance knowledge may effectively improve both attitudes and practical behaviors, though more rigorous research is needed to confirm these relationships.

## Introduction

Co-infection with human immunodeficiency virus (HIV) and tuberculosis (TB) constitutes a significant global public health challenge [1]. Tuberculosis remains the leading cause of mortality among individuals living with HIV, accounting for nearly 30% of Acquired Immunodeficiency Syndrome (AIDS)-related deaths worldwide in 2019 [2]. The magnitude of this dual epidemic is substantial; in 2021, an estimated 1.76 million individuals were co-infected with HIV and TB globally, resulting in approximately 200,895 fatalities [3]. Individuals diagnosed with HIV exhibit a markedly increased susceptibility to the development of active TB in comparison to their HIV-negative counterparts, and this heightened risk persists even among patients undergoing antiretroviral therapy [4,5].

The epidemiological landscape in China indicates evolving patterns of HIV-TB coinfection. From 2015 to 2019, the prevalence of HIV-TB coinfection among TB patients increased from 0.8% to 1.1%. Conversely, the proportion of HIV-positive individuals with TB declined from 1.1% to 0.8% over the same period [6]. In Beijing, the coinfection rate remains relatively high, approximately 1–2% [7]. Despite these seemingly modest statistics, the management of HIV-TB coinfection poses significant challenges, including potential drug-drug interactions between rifampicin and antiretroviral agents, a substantial burden of opportunistic infections, and the escalating threat of multidrug-resistant TB [8,9]. Effective management of this co-infection is crucial, as it profoundly impacts patient survival and quality of life, underscoring the necessity for a well-informed and proficient healthcare workforce.

While numerous studies have employed the KAP framework to assess healthcare providers’ competencies, most remain at the descriptive level, failing to elucidate the specific transformation pathways from knowledge acquisition to behavioral change. Understanding these mechanistic relationships is crucial for developing precision-targeted training interventions, particularly in complex clinical scenarios such as HIV/TB co-infection management. The Knowledge, Attitude, and Practice (KAP) framework provides a structured model for understanding behavioral change. This model posits that knowledge forms the foundation upon which attitudes and beliefs develop, ultimately driving behavioral transformation [10]. The progression typically occurs in sequential stages: knowledge acquisition, attitude formation, and behavior adoption [11]. It is important to note, however, that behavior change depends not solely on knowledge acquisition but also on fundamental shifts in perception [11].

Existing literature indicates that HCPs often lack comprehensive knowledge about TB prevention and control, with only half possessing sufficient knowledge, and even fewer consistently adhering to protective measures [12–17]. Moreover, while awareness of post-exposure prophylaxis (PEP) is high, actual adherence following potential exposures remains low [18]. These findings suggest that HCPs may not be adequately prepared to handle the complexities of HIV-TB co-infection, which requires integrated knowledge of both diseases and their interactions.

This study aimed to reveal, for the first time, the specific transformation pathways and obstacle nodes from knowledge to practice among healthcare providers in HIV/TB co-infection management using structural equation modeling (SEM), thereby providing empirical evidence for precision training strategies. In particular, the study sought to identify not only the direct relationships among knowledge, attitudes, and practices but also the mediating mechanisms and barriers that hinder the translation of knowledge into practice in HIV/TB co-infection management. To this end, we conducted a multicenter cross-sectional survey of healthcare providers in Beijing and applied SEM to examine both the direct and indirect effects of knowledge on practice through attitudes, with the goal of informing targeted training interventions for HIV/TB co-infection care.

## Materials and methods

### Study design, setting and participants

This multicenter cross-sectional study engaged HCPs from various medical institutions in Beijing between May and June, 2025. The participating sites included several hospitals affiliated with Capital Medical University, such as Beijing Ditan Hospital, Beijing You’an Hospital, Beijing Shijitan Hospital, and Beijing Chaoyang Hospital, in addition to other prominent tertiary hospitals, including the Fifth Medical Center of the Chinese PLA General Hospital, the First Affiliated Hospital of Tsinghua University, Peking University Third Hospital, and Beijing Geriatric Hospital, along with several community hospitals. Inclusion Criteria: Certified physicians or registered nurses employed at either tertiary or community hospitals, as well as HCPs who provided voluntary informed consent to participate in the study. No exclusion criteria were established. Ethical approval for the study was granted by the Ethics Committee of Beijing Ditan Hospital, Capital Medical University (Approval NO. NO.DTEC-KY2025-060-01). All participants provided informed consent prior to their involvement in the study.

### Questionnaire

The KAP questionnaire (Electronic Questionnaire) was developed based on expert consensus regarding the diagnosis and treatment of co-infection with HIV and Mycobacterium tuberculosis [19], as well as insights derived from previously published studies [20,21]. The questionnaire comprises four distinct dimensions: 1) Demographic Information: This section encompasses variables such as gender, age, and professional position. 2) Knowledge Dimension: This dimension consists of 15 items, each rated on a scale wherein participants receive 2 points for “very familiar,” 1 point for “heard of,” and 0 points for “unclear.” The cumulative score ranges from 0 to 30 points. 3) Attitude Dimension: This section includes 10 items, assessed using a five-point Likert scale ranging from “strongly agree” to “strongly disagree.” Scores are assigned from 5 to 1, resulting in a potential total score between 10 and 50 points. 4) Practice Dimension: This component comprises 8 items, rated from 5 to 1 based on responses that range from “always” to “never,” yielding a score range of 8–40 points. Scores that exceed 75% of the maximum possible in each dimension are indicative of adequate knowledge, a favorable attitude, and proactive practices [22]. A small pre-test (44 copies) was conducted before the formal launch, and Cronbach’s α was 0.962, suggesting a satisfactory internal consistency. The use of self-administered questionnaires to assess KAP has been widely validated in infectious disease research, including HIV and tuberculosis studies, as an effective approach to capture provider awareness and behavioral intent while maintaining feasibility in large samples [23].

### Questionnaire distribution

Online e-questionnaires were administered via the Sojump platform (https://www.wjx.cn/) in China. A convenience sampling method was employed to select HCPs. Upon confirming eligibility according to the established inclusion criteria and securing informed consent for participation, a QR code was provided to facilitate access to the electronic questionnaire. To mitigate the risk of duplicate responses, an IP address restriction was implemented, thereby permitting each survey to be completed only once per IP address. Following data collection, the research team conducted a quality assessment of the completed questionnaires. Responses submitted in under 90 seconds, as well as those exhibiting clear logical inconsistencies or repetitive answer patterns, were classified as invalid and consequently excluded from the analysis. We acknowledge that using a self-administered questionnaire relies on participants’ recall and may introduce recall bias; however, this risk was minimized through pre-testing, response-quality screening, and consistency checks during data validation.

### Sample size

Sample size was calculated using the formula for a cross-sectional study [24]: α = 0.05, $\text{n}={\left(\frac{Z_{\text{1}-\alpha /\text{2}}}{\delta }\right)}^{\text{2}}\times p\times (\text{1}-p)$ where $Z_{\text{1}-\alpha /\text{2}}$=1.96 when α = 0.05, the assumed degree of variability of p = 0.5 maximises the required sample size, and δ is admissible error (which was 5% here). The theoretical sample size was 480 which includes an extra 20% to allow for subjects lost during the study.

### Statistical analysis

Statistical analysis was conducted utilizing the Statistical Package for the Social Sciences (SPSS) version 27.0 and Analysis of Moment Structures (AMOS) version 26.0, both developed by IBM Corp., Armonk, NY, USA. The continuous data were tested for normality using the Kolmogorov–Smirnov test. Normally distributed continuous data were expressed as means ± standard deviations (SD) and analyzed using Student’s t-test (two levels) or ANOVA (more than two levels). Continuous data with skewed distribution were presented as medians (ranges) and analyzed using the Mann–Whitney U-test (two levels) or the Kruskal–Wallis test (more than two levels). Categorical variables are expressed as n (%). The correlations between KAP dimension scores were assessed using Spearman correlation analysis, as the KAP scores were ordinal in nature and did not fully satisfy the normality assumption required for Pearson correlation. Spearman’ s rank correlation is a non-parametric method that evaluates monotonic relationships between variables, making it more appropriate for skewed or non-normally distributed data in this study. Furthermore, a SEM analysis was executed to investigate the interrelationships among KAP. SEM was selected because it allows simultaneous testing of multiple dependent relationships and the estimation of both direct and indirect effects among latent constructs, which is particularly suitable for exploring the pathways through which knowledge and attitudes influence practice in the KAP framework. Model fit was evaluated utilizing established indices, including the Chi-square to degrees of freedom ratio (CMIN/DF), Root Mean Square Error of Approximation (RMSEA), Incremental Fit Index (IFI), Tucker-Lewis Index (TLI), and Comparative Fit Index (CFI). The hypotheses for the SEM posited that: (1) knowledge directly influences attitude, (2) attitude directly influences practice, and (3) knowledge exerts both direct and indirect influences on practice [25]. All statistical analyses were conducted with a two-sided test, and a P value of less than 0.05 was deemed statistically significant. Additionally, the internal consistency and construct validity of the KAP questionnaire were examined. Cronbach’s alpha values were calculated for each item and domain, and standardized factor loadings were obtained (Supplementary Table S1 and S2 in S2 Appendix).

## Results

### Demographic characteristics and KAP scores

A total of 588 questionnaires were initially collected for this study. However, 21 responses were excluded due to a response time of less than 90 seconds, and 2 were excluded because the respondents were under 18 years of age. This resulted in a final count of 565 valid questionnaires, yielding an effective response rate of 96.09%. Among the respondents, 79.12% were female, with a mean age of 36.88 ± 8.84 years. The overall Cronbach’s α coefficient for the questionnaire was 0.953, and the Kaiser–Meyer–Olkin (KMO) measure of sampling adequacy was 0.950 (P < 0.001), indicating strong internal consistency and suitability for further statistical analysis. Physicians constituted the majority of respondents (64.42%), with the majority working in tertiary hospitals (62.12%) and 37.88% engaged in infectious disease-related roles, such as infection management.

**Fig 1:**
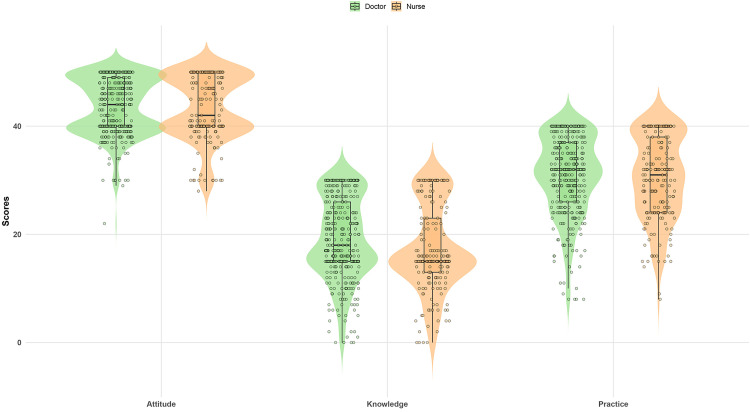
Distribution of Knowledge, Attitude, and Practice Scores among Doctors and Nurses.

The knowledge, attitude, and practice scores were 18.51 ± 7.75 (possible range: 0–30), 43.64 ± 5.51 (possible range: 10–50), and 30.75 ± 7.44 (possible range: 8–40), respectively. When comparing the two primary professional roles, physicians exhibited significantly higher knowledge scores than nurses (P = 0.002). However, no statistically significant differences were observed in attitude (P = 0.810) or practice scores (P = 0.628) between the two groups. These findings indicate a generally comparable performance in these domains across both physicians and nurses (Table 1, Fig 1). The internal consistency of the questionnaire was strong, with Cronbach’s alpha values for individual items ranging from 0.590 to 0.857 across the KAP domains (Supplementary Table S1 in S2 Appendix). All standardized factor loadings exceeded 0.59 (P < 0.001), supporting the construct validity of the questionnaire (Supplementary Table S2 in S2 Appendix).

**Table 1:** Baseline Characteristics.

Variables	N (%)	Knowledge, mean ± SD	*P*	Attitude, mean ± SD	*P*	Practice, mean ± SD	*P*
**N = 565**		18.51 ± 7.75		43.64 ± 5.51		30.75 ± 7.44	
**Gender**			0.257		0.479		0.848
Male	118 (20.88)	19.24 ± 7.12		43.28 ± 5.44		30.91 ± 7.11	
Female	447 (79.12)	18.32 ± 7.90		43.74 ± 5.53		30.71 ± 7.53	
**Age**	36.88 ± 8.84						
**Position**			0.002		0.810		0.628
Doctor	364 (64.42)	19.18 ± 7.56		43.73 ± 5.28		30.89 ± 7.28	
Nurse	201 (35.58)	17.29 ± 7.95		43.49 ± 5.90		30.50 ± 7.73	
**Nature of institution**			0.010		0.689		0.633
Tertiary hospital	351 (62.12)	19.32 ± 7.51		43.65 ± 5.34		31.05 ± 7.07	
Community hospitals	173 (30.62)	17.12 ± 8.20		43.47 ± 5.84		30.16 ± 8.02	
Other	41 (7.26)	17.49 ± 6.99		44.32 ± 5.59		30.66 ± 8.02	
**Department**			<0.001		0.177		0.194
Community Hospital Infectious Disease Related Work (Infectious Disease Nursing/Hospital Infection Management)	214 (37.88)	20.77 ± 7.72		44.25 ± 5.20		31.58 ± 6.93	
Basic Medicine/Administration/Graduate student	93 (16.46)	17.15 ± 6.38		43.48 ± 5.08		30.37 ± 7.99	
Non-Infectious Disease Nursing	258 (45.66)	17.13 ± 7.81		43.20 ± 5.87		30.20 ± 7.60	
**Educational background**			<0.001		0.809		0.317
Technical secondary school/Associate degree	69 (12.21)	18.67 ± 8.02		43.38 ± 6.64		31.58 ± 7.35	
Bachelor’s degree	267 (47.26)	17.15 ± 7.90		43.49 ± 5.58		30.18 ± 7.78	
Master’s degree or above	229 (40.53)	20.04 ± 7.21		43.91 ± 5.04		31.16 ± 7.03	
**Years of work experience**			0.920		0.762		0.407
≤5 years	139 (24.6)	18.83 ± 7.53		43.41 ± 5.43		31.34 ± 7.00	
5-10 years	109 (19.29)	18.43 ± 7.72		43.97 ± 5.30		31.07 ± 7.81	
11-15 years	128 (22.65)	18.73 ± 7.90		43.66 ± 6.14		30.75 ± 7.99	
≥16 years	189 (33.45)	18.17 ± 7.87		43.61 ± 5.26		30.13 ± 7.16	
**Professional title**			0.006		0.242		0.039
None/junior	220 (38.94)	18.18 ± 7.75		43.75 ± 5.78		31.38 ± 7.71	
Intermediate	219 (38.76)	17.70 ± 7.70		43.21 ± 5.59		29.80 ± 7.59	
Associate senior/senior	126 (22.30)	20.50 ± 7.55		44.21 ± 4.82		31.29 ± 6.52	
**Family member with pulmonary tuberculosis**			0.789		0.097		0.371
Yes	22 (3.89)	18.73 ± 6.27		42.27 ± 4.50		29.82 ± 5.31	
No	543 (96.11)	18.50 ± 7.81		43.70 ± 5.54		30.79 ± 7.51	
**Colleagues with pulmonary tuberculosis**			0.003		0.187		0.899
Yes	91 (16.11)	20.70 ± 7.13		44.46 ± 4.95		30.88 ± 7.04	
No	474 (83.89)	18.09 ± 7.80		43.49 ± 5.60		30.73 ± 7.52	
**Number of tuberculosis patients handled**			<0.001		0.006		0.014
5 or fewer	374 (66.19)	17.07 ± 7.52		43.18 ± 5.74		30.14 ± 7.63	
5-20	98 (17.35)	20.11 ± 7.59		43.92 ± 4.79		31.42 ± 6.88	
More than 20	93 (16.46)	22.61 ± 7.06		45.22 ± 5.00		32.51 ± 6.93	
**Training related to Mycobacterium tuberculosis**			<0.001		<0.001		<0.001
Yes	409 (72.39)	20.26 ± 7.46		44.26 ± 5.32		31.91 ± 7.35	
No	156 (27.61)	13.92 ± 6.54		42.04 ± 5.68		27.71 ± 6.81	

Knowledge scores varied significantly based on education level, professional role, annual management of TB patients, TB-related training, and professional title (all P < 0.01). Individuals with a master’s degree or higher, those in infectious disease positions, those managing over 20 TB cases annually, and those who had undergone TB-related training had significantly higher knowledge scores. Advanced professional titles were also positively correlated with knowledge. Attitude scores were significantly associated with the number of TB cases managed (P = 0.006) and participation in TB-related training (P < 0.001), indicating that exposure to TB patients and relevant training positively influenced attitudes. No significant correlation was found with professional role (P = 0.177). Practice scores showed significant associations with the number of TB patients managed, TB training, and professional title. Higher practice scores were noted among professionals with substantial clinical exposure to TB patients and those who had received specialized training, with senior professionals exhibiting particularly high practice scores (Table 1).

### Distribution of knowledge, attitude, and practice dimension

The distribution of knowledge dimensions showed that the three questions with the highest number of participants choosing the ‘Unclear’ option were “Treatment failure in drug-susceptible tuberculosis refers to cases where the patient remains culture-positive after four months of anti-TB treatment” (**K14**) with 23.89%, “For all newly diagnosed TB patients, routine drug susceptibility testing for first-line anti-tuberculosis drugs is recommended. For patients who remain culture-positive after four months of treatment or who become culture-positive again after initial conversion, repeated first-line drug susceptibility testing is recommended.” (**K13**) with 22.65%, and “The treatment of multidrug-resistant TB (MDR-TB) and extensively drug-resistant TB (XDR-TB) should be individualized, taking into account the drug resistance pattern of MTB, the availability of anti-TB drugs, disease severity, and co-infections.” (**K15**) with 22.48% (Supplementary Table S3 in S2 Appendix). Responses to the attitude dimension showed that while most participants demonstrated positive attitudes toward HIV/TB co-infection management, notable gaps emerged in specific areas. A pronounced concern was the perceived inadequacy of institutional training: 67.79% of HCPs (31.15% strongly agreeing, 36.64% agreeing) indicated that hospital-based training on HIV/TB co-infection and drug resistance was insufficient (**A10**), with an additional 24.96% remaining neutral. Suboptimal enthusiasm was also observed toward clinical innovations, as 16.28% expressed neutrality and 1.24% disagreed with interest in new diagnostic technologies (**A6**), while 18.05% were neutral and 1.77% disagreed regarding updates to treatment regimens (**A7**) (Supplementary Table S4 in S2 Appendix). Responses to the practice dimension showed that 16.11% rarely and 11.68% never recommend drug resistance gene testing for patients with HIV co-infected with tuberculosis (**P1**), 17.7% rarely and 7.26% never educate tuberculosis patients about methods and benefits of drug-resistant tuberculosis testing (**P2**), 13.1% rarely and 6.73% never monitor patients’ drug resistance test results and adjust treatment accordingly (**P8**) (Supplementary Table S5 in S2 Appendix).

### Spearman correlations analysis

Spearman correlation analysis revealed positive correlations between knowledge and attitude (r = 0.500, P < 0.001), as well as between knowledge and practice (r = 0.592, P < 0.001). Additionally, attitude was positively correlated with practice (r = 0.584, P < 0.001) (Table 2).

**Table 2:** Spearman correlation analysis.

Dimensions	Knowledge	Attitude	Practice
**Knowledge**	1		
**Attitude**	0.500 (P < 0.001)	1	
**Practice**	0.592 (P < 0.001)	0.584 (P < 0.001)	1

### Structural equation model analysis

**Fig 2:**
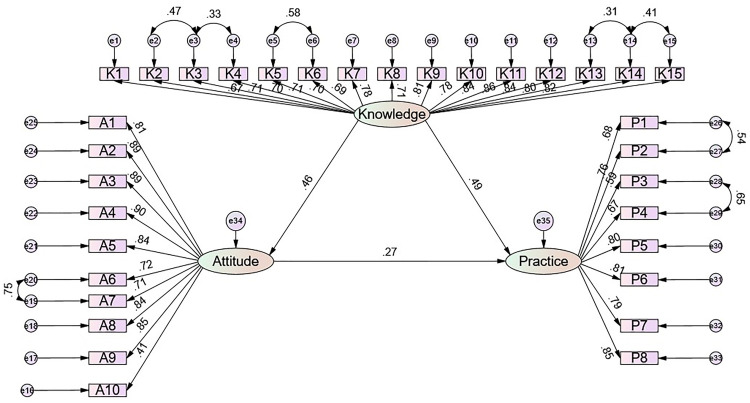
Standardized SEM model.

The SEM demonstrated a highly favorable model fit indices (CMIN/DF value: 4.460, RMSEA value: 0.078, IFI value: 0.900, TLI value: 0.891, and CFI value: 0.900), suggesting a well-fitting model (Supplementary Table S6 in S2 Appendix), and the effect estimates between the various paths have been presented (Supplementary Table S2 in S2 Appendix), and the SEM results showed that the direct effect of knowledge on both attitude (β = 0.458, P = 0.002) and practice (β = 0.491, P = 0.011), as well as of attitude on practice (β = 0.272, P = 0.008), furthermore, knowledge indirectly affected practice through attitude (β = 0.124, P = 0.005) (Table 3). All observed indicators loaded strongly onto their respective latent constructs, with standardized factor loadings consistently above 0.58 across the knowledge (K1–K15), attitude (A1–A10), and practice (P1–P8) domains, supporting the measurement validity of the model. The diagram also visually confirms that knowledge exerts the strongest direct path to practice, while attitude provides an additional mediating pathway, consistent with the numerical SEM estimates (Fig 2).

**Table 3:** Structural equation model analysis.

Model paths	Standardized Total effects		Standardized direct effects		Standardized indirect effects	
	β (95%CI)	P	β (95%CI)	P	β (95%CI)	P
Knowledge→Attitude	0.458 (0.397-0.556)	0.002	0.458 (0.397-0.556)	0.002		
Knowledge→Practice	0.615 (0.554-0.673)	0.007	0.491 (0.406-0.574)	0.011		
Attitude→Practice	0.272 (0.169-0.353)	0.008	0.272 (0.169-0.353)	0.008		
Knowledge→Practice					0.124 (0.079-0.171)	0.005

## Discussion

Our sample revealed insufficient knowledge yet maintained generally positive attitudes and moderate practices regarding HIV and TB co-infection. Critically, the SEM analysis demonstrated that the direct effect of knowledge on practice substantially exceeded the indirect effect mediated through attitude, suggesting that knowledge deficits constitute the primary bottleneck in behavioral transformation. This finding indicated that even when HCPs maintain positive attitudes, insufficient knowledge, particularly regarding drug resistance testing protocols, directly impedes evidence-based practices.

This study revealed a notable misalignment between the relatively positive attitudes and only moderate practices among healthcare professionals in Beijing when it comes to HIV/TB co-infection, largely underpinned by insufficient knowledge. Although many respondents expressed general agreement on the importance of prevention and expressed interest in updated diagnostics and treatments, their ability to accurately identify key clinical and diagnostic principles, such as the use of molecular techniques or the complexity of co-infection in immunocompromised patients, remained limited. This knowledge gap has been consistently observed in other settings, including studies conducted in urban and semi-urban Chinese provinces, where high disease burden does not always correspond with high clinical preparedness [26,27]. The gap between awareness and behavior is particularly concerning in the context of HIV and TB co-infection, which is well-documented to complicate clinical management and increase mortality risk if not managed comprehensively [28]. A noteworthy finding was that despite HCPs in tertiary hospitals demonstrating superior knowledge scores, this advantage did not translate into significantly better practice scores. It is noteworthy that only 37.88% of respondents were engaged in infectious-disease related roles. This may reflect the multidisciplinary nature of HIV/TB co-infection management, which often involves general physicians, nurses in non-specialist departments, and health workers in primary/community settings. Previous qualitative work in high HIV/TB burden settings shows that providers whose roles are not dedicated to infectious disease tend to have less frequent exposure to co-infection cases and less training in integrated care, which may lead to lower practice scores [29,30]. This dissociation may reflect systemic barriers including heavy clinical workloads, inadequate incentive mechanisms, and organizational constraints, suggesting that knowledge enhancement alone is insufficient for behavioral change without addressing structural impediments.

The positive associations observed between knowledge, attitudes, and practices suggest that strengthening HCPs’ knowledge base could facilitate more consistent behavior and improve adherence to protocols. This pathway is supported by our structural equation model, where knowledge was shown to directly influence both attitudes and practices, and also indirectly affect practices through changes in attitude. These findings echo conclusions from previous studies that emphasized the importance of integrated training interventions to improve co-infection care [31,32].

Disparities in performance based on institutional affiliation and educational background further reflect structural inequities. Disparities in performance based on institutional affiliation and educational background further reflect structural inequities. Integrated training interventions refer to educational programmes that combine content on both HIV and TB. including co-infection, diagnosis, treatment, and prevention. into a unified curriculum rather than addressing each disease separately. Such integration enables healthcare providers to understand the synergistic interactions between HIV and TB, streamline decision-making, and reduce fragmentation of care. Evidence from recent implementation studies demonstrates that co-locating HIV and TB services and synchronising provider training can improve clinical preparedness, teamwork, and patient outcomes [33,34]. Within the KAP framework, integrated training can strengthen knowledge by presenting both HIV and TB content coherently, enhance attitudes by reinforcing the value of coordinated management, and improve practice through case-based exercises and workflow simulations that reflect real co-infection scenarios. These programmes therefore support the direct and indirect pathways from knowledge to practice identified in our SEM model.

Those affiliated with tertiary care centers and holding higher academic qualifications generally scored better in knowledge, although this advantage did not translate as clearly into improved practices. Similar patterns have been noted in previous research, where higher-tier institutions have greater access to training and resources but may not ensure that this knowledge permeates through routine clinical workflows [35]. The analysis revealed no statistically significant differences in KAP performance, particularly concerning attitude and practice, between physicians and nurses. This suggests that professional role may not be the predominant factor influencing competency in managing HIV/TB co-infection within this context. Rather, specialized training, direct clinical exposure to tuberculosis cases, and institutional support appear to play a more critical and equitable role across these professions. Furthermore, clinical experience with TB cases and prior participation in targeted training were identified as the most consistent determinants of elevated KAP scores. These findings underscore the importance of experiential learning, which prior research has indicated enhances the retention and practical application of knowledge related to infectious diseases [36].

The distribution of KAP dimensions reveals significant content-specific deficiencies. While respondents generally acknowledged the broader public health implications of HIV/TB co-infection, their familiarity with emerging diagnostic technologies, mechanisms of drug resistance, and the complexities of managing extrapulmonary presentations were notably limited. In particular, knowledge items pertaining to molecular diagnostics and the interpretation of resistance profiles received the weakest responses, which corresponds with previous research that underscores the necessity for improved laboratory-clinical integration within training curricula [37].

Several factors may explain the observed low knowledge levels among HCPs regarding HIV/TB co-infection. First, many healthcare providers receive only general infectious-disease training, with limited exposure to integrated HIV/TB modules in undergraduate or continuing medical education. For example, a Chinese study of health professionals found that correct responses on HIV/AIDS knowledge ranged from only 3% to 68% and that attendance at workshops did not guarantee adequate understanding [38]. Second, providers in non-specialist roles or settings with fewer HIV/TB cases have less practical exposure, which correlates with lower knowledge levels, as seen in settings where non-NTP (national tuberculosis programme) providers had significantly lower TB knowledge scores [39]. These findings suggest that the educational and experiential foundations for managing HIV/TB co-infection may be weaker than for either disease alone, contributing to the knowledge deficits identified in our study.

Although attitudes were predominantly positive, they also indicated subtle concerns regarding institutional support for training and resources. This finding aligns with studies conducted in other middle-income countries, wherein professional willingness frequently exceeds institutional capacity [40].

Practices remained variable and appeared to lag behind attitudes. Although most participants reported fulfilling basic patient education responsibilities and initial risk assessments, more advanced practices such as proactive testing recommendations or personal engagement in continuous learning were less common. These behaviors, while influenced by personal initiative, are also shaped by institutional expectations and resource availability. A number of studies have emphasized that without enabling systems, even motivated providers may be constrained in their ability to act [41]. The disconnect between individual commitment and system-level support continues to pose a challenge for effective implementation of co-infection protocols.

The findings from this study point to several implications for policy and practice. To begin with, institutions Our preliminary findings suggest that institutions may benefit from reviewing co-infection management protocols to ensure they are accessible and consistently applied across departments. Efforts should be made to develop routine training programs that are tailored to different staff categories, particularly for nurses and community-based providers who may have less exposure to specialist content. Training should move beyond general knowledge delivery to include case-based simulations, guideline application workshops, and mentoring programs, which have been shown to improve practical skills in similar clinical areas [42]. These results provide initial evidence that health authorities might consider exploring mechanisms to formally integrate HIV/TB modules into continuing medical education requirements and link training participation to professional advancement criteria.

At a broader level, resource allocation must be improved to ensure that diagnostic and protective tools needed for co-infection management are readily available in both tertiary and primary care settings. Collaboration with national tuberculosis and HIV programs can help harmonize messaging and ensure consistency in practice expectations. Furthermore, strategies should be adapted to reflect the unique constraints of different healthcare environments. In community hospitals, where staffing and laboratory capacity may be limited, simplified protocols and digital decision-support tools may be particularly beneficial. In contrast, tertiary hospitals might benefit more from advanced workshops focusing on new diagnostic platforms and complex case discussions. Sustainability of these interventions will require not only financial support but also institutional leadership and long-term planning [43,44]. The findings from this study may serve as an evidence base for the development of standardized training programs and targeted interventions to improve clinical care and prevention of HIV/TB co-infection.

It should be noted that in China, although HIV/AIDS prevention and TB control are both incorporated into continuing medical education and public‐health training, published information on the systematic inclusion of HIV/TB co‐infection in undergraduate medical curricula is scarce. A recent meta-analysis of HIV/TB coinfection in mainland China called for enhanced screening and integration of services, highlighting a potential gap in educational preparation [45]. Therefore, our findings of limited knowledge among healthcare providers may partially reflect a broader lack of formal training in this specific co-infection. Future curriculum development in Chinese medical schools and postgraduate training programmes should consider incorporating HIV/TB coinfection content explicitly.

This study has several important limitations. First, convenience sampling was employed, which may introduce significant selection bias as participants voluntarily responding to the survey might have greater interest in HIV/TB co-infection topics and higher baseline knowledge levels compared to the general HCP population. Convenience sampling was adopted instead of consecutive sampling due to practical constraints across multiple institutions, where varying administrative processes and workloads made consecutive recruitment unfeasible. This approach is commonly applied in cross-sectional KAP studies to identify association patterns rather than population estimates. This sampling method severely limits the generalizability of findings to all HCPs in Beijing. Second, as a cross-sectional survey, it captures associations at a single time point and cannot establish causal relationships between knowledge, attitudes, and practices. Third, the reliance on self-reported data may introduce social desirability bias, potentially leading participants to overreport favorable attitudes or practices.

## Conclusion

In our sample, respondents demonstrated insufficient knowledge, relatively positive attitudes, and moderate levels of practical engagement concerning HIV/TB co-infection, with knowledge exerting both direct and indirect effects on practice through attitudes. These findings highlight the need for targeted educational interventions to enhance knowledge among HCPs, which may, in turn, strengthen their attitudes and practices in managing HIV/TB co-infection.

## Supporting information

S1 Appendix Electronic Questionnaire. (DOCX)

S2 Appendix Supplementary Tables: Supplementary Table 1. Cronbach’s alpha coefficients for individual KAP items. Supplementary table 2. Estimated total effect coefficient. Supplementary table 3. Distribution of knowledge dimension responses. Supplementary table 4. Distribution of attitude dimension responses. Supplementary table 5. Distribution of practice dimension responses. Supplementary table 6. SEM fit indicators.
(DOCX)
