# Supplementary material for: Knowledge, attitudes, and practices of healthcare providers in Beijing regarding human immunodeficiency virus and tuberculosis co-infection: A multicenter cross-sectional study
Source: PLoS One. 2026 Feb 23;21(2):e0341132. doi: 10.1371/journal.pone.0341132 (PMC12928499; doi:10.1371/journal.pone.0341132)
Supplement: S1 Appendix — (DOCX) [file pone.0341132.s001.docx]

**Appendix 1 Electronic Questionnaire**

| Questionnaire ID： |
| --- |
| Dear Participant,  We are researchers from XXX and sincerely invite you to participate in our study. This research aims to assess the knowledge, attitudes, and practices of healthcare professionals in Beijing regarding HIV/TB co-infection, providing a scientific basis for developing effective intervention strategies. These strategies may help more people and improve their health outcomes in the future. Your participation is entirely voluntary. This study has been reviewed and approved by the Ethics Review Committee. If you agree to participate, please read the following information:  1. Please complete the questionnaire. There are no right or wrong answers—simply answer based on your actual situation. If you encounter any questions during the process, feel free to ask us. Please submit the questionnaire once completed.  2. This is a simple questionnaire survey that will not cause any physical or psychological harm. However, it includes some personal questions such as your gender and age. We will strictly protect your privacy and ensure your information is kept confidential.  3. As a participant, you have the right to access information and updates related to the study at any time. If you decide to withdraw from the study, please inform us, and your data will not be included in the analysis.  Finally, we sincerely thank you for taking the time out of your busy schedule to support our scientific research!  □I have read and agree that the data collected may be used for scientific research.  Signature of Informed Consent:  Date of Participation: Year Month Day |

| **Part 1 Basic Information** | | |
| --- | --- | --- |
| **1. Your gender:** | a. Male | b. Female |
| **2. Your age: 。** | | |
| **3. Your position:** a. Doctor b. Nurse | | |
| **4. The institution you work for: (  ) (Please select from the target hospitals listed in the Questionnaire Star system)** | | |
| **5. Your type of healthcare professional:**  a. Infectious disease department doctor or nurse | | |
| b. Hospital infection control staff | | |
| c. Basic medical science researcher | | |
| d. Hospital administrative staff | | |
| e. Medical graduate student | | |
| f. Non-infectious disease department doctor or nurse | | |
| **6. Your educational background:** | a. Technical secondary school/Associate degree b. Bachelor's degree c. Master's degree or above | |
| **7. Your years of work experience:** | a. ≤5 years b. 5–10 years c. 11–15 years d. ≥16 years | |
| **8. Nature of your institution:** | a. Public primary/secondary hospital b. Public tertiary hospital c. Private hospital | |
| **9. Your professional title:** | a. None b. Junior c. Intermediate d. Associate senior e. Senior | |
| **10. Have any of your family members (parents, children, or siblings) ever been diagnosed with pulmonary tuberculosis?** | a. Yes  b. No | |
| **11. Have any of your colleagues ever been diagnosed with pulmonary tuberculosis?** | a. Yes  b. No | |
| **12. How many tuberculosis patients have you handled?** | a. 5 or fewer b. 5–10 c. 11–20 d. More than 20 | |
| **13.** **Have you received any training related to Mycobacterium tuberculosis and drug-resistant TB infection?** | a. Yes  b. No | |

**Part 2 Knowledge of HIV/M. Tuberculosis Co-Infection and Drug Resistance**

| 1. After being infected with *Mycobacterium tuberculosis*, the human body may develop either latent tuberculosis infection or active tuberculosis. | a. Very familiar | b. Heard of it | c. Unclear |
| --- | --- | --- | --- |
| 2. HIV infection is an independent risk factor for developing tuberculosis, and people living with HIV have a significantly higher risk of latent tuberculosis infection progressing to active TB compared to those without HIV. | a. Very familiar | b. Heard of it | c. Unclear |
| 3. Tuberculosis is one of the most common opportunistic infections among people living with HIV. It is a major factor in disease progression and a leading cause of death in AIDS patients. | a. Very familiar | b. Heard of it | c. Unclear |
| 4. With the widespread use of anti-tuberculosis drugs, the emergence and spread of drug-resistant tuberculosis has become a global issue. | a. Very familiar | b. Heard of it | c. Unclear |
| 5. Prompt identification of Mycobacterium tuberculosis infection is key to early clinical diagnosis and treatment. | a. Very familiar | b. Heard of it | c. Unclear |
| 6. The gold standard for clinical diagnosis of *Mycobacterium tuberculosis* is the isolation of the organism from secretions or fluids (e.g., sputum, bronchoalveolar lavage fluid, or pleural effusion) or from tissues (e.g., pleural biopsy or lung biopsy), or a positive nucleic acid identification. | a. Very familiar | b. Heard of it | c. Unclear |
| 7. The diagnosis of HIV/MTB co-infection is relatively more difficult, with atypical clinical manifestations. The presence of multiple other opportunistic infections complicates the condition, and extrapulmonary tuberculosis is relatively more common. | a. Very familiar | b. Heard of it | c. Unclear |
| 9. Tuberculin skin tests and interferon-gamma release assays are specifically used for the diagnosis of tuberculosis infection. | a. Very familiar | b. Heard of it | c. Unclear |
| 10. Molecular testing, such as gene probes and sequencing, can be used to detect Mycobacterium tuberculosis DNA and common mutations associated with drug resistance. | a. Very familiar | b. Heard of it | c. Unclear |
| 11. Tuberculosis infection may occur in HIV-positive individuals regardless of their CD4+ T lymphocyte count. | a. Very familiar | b. Heard of it | c. Unclear |
| 12. High-throughput sequencing is widely used in clinical practice for the diagnosis of tuberculosis infection and the detection of drug resistance. | a. Very familiar | b. Heard of it | c. Unclear |
| 13. The treatment regimen for tuberculosis in people living with HIV is the same as for those with tuberculosis alone, but drug interactions between anti-TB and antiretroviral medications must be considered. | a. Very familiar | b. Heard of it | c. Unclear |
| 14. For all newly diagnosed TB patients, routine drug susceptibility testing for first-line anti-tuberculosis drugs is recommended. For patients who remain culture-positive after four months of treatment or who become culture-positive again after initial conversion, repeated first-line drug susceptibility testing is recommended. | a. Very familiar | b. Heard of it | c. Unclear |
| 15. Treatment failure in drug-susceptible tuberculosis refers to cases where the patient remains culture-positive after four months of anti-TB treatment (five months according to China, Europe, and WHO definitions). | a. Very familiar | b. Heard of it | c. Unclear |
| 16. The treatment of multidrug-resistant TB (MDR-TB) and extensively drug-resistant TB (XDR-TB) should be individualized, taking into account the drug resistance pattern of MTB, the availability of anti-TB drugs, disease severity, and co-infections. | a. Very familiar | b. Heard of it | c. Unclear |

| **Part 3 Attitudes Toward HIV Co-Infection with Mycobacterium Tuberculosis and Drug Resistance** | | | | | |
| --- | --- | --- | --- | --- | --- |
| 1. You believe that HIV co-infection with Mycobacterium tuberculosis and drug resistance poses a significant threat to public health. | a. strongly agree | b. agree | c. neutral | d. disagree | e. strongly disagree |
| 2. You believe it is important to be well-versed in knowledge related to HIV co-infection with Mycobacterium tuberculosis and drug resistance. | a. strongly agree | b. agree | c. neutral | d. disagree | e. strongly disagree |
| 3. You believe that HIV co-infection with Mycobacterium tuberculosis and drug resistance is a complex condition. | a. strongly agree | b. agree | c. neutral | d. disagree | e. strongly disagree |
| 4. You believe it is important to implement protocols to prevent the transmission of HIV co-infection with Mycobacterium tuberculosis and drug resistance. | a. strongly agree | b. agree | c. neutral | d. disagree | e. strongly disagree |
| 5. You believe that the diagnosis of HIV co-infection with Mycobacterium tuberculosis and drug resistance should strictly follow guidelines. | a. strongly agree | b. agree | c. neutral | d. disagree | e. strongly disagree |
| 6. You are very interested in new diagnostic technologies for HIV co-infection with Mycobacterium tuberculosis and drug resistance. | a. strongly agree | b. agree | c. neutral | d. disagree | e. strongly disagree |
| 7. You are very interested in updates to treatment regimens for HIV co-infection with Mycobacterium tuberculosis and drug resistance. | a. strongly agree | b. agree | c. neutral | d. disagree | e. strongly disagree |
| 8. You believe that managing patients with HIV co-infection and drug-resistant Mycobacterium tuberculosis is a challenging but meaningful task. | a. strongly agree | b. agree | c. neutral | d. disagree | e. strongly disagree |
| 9. You believe that increasing awareness of HIV co-infection with Mycobacterium tuberculosis and drug resistance can reduce the risk of transmission. | a. strongly agree | b. agree | c. neutral | d. disagree | e. strongly disagree |
| 10. You believe that the training provided by hospitals on HIV co-infection with Mycobacterium tuberculosis and drug resistance is insufficient. | a. strongly agree | b. agree | c. neutral | d. disagree | e. strongly disagree |

| **Part 4 Practices Regarding HIV Co-Infection with Mycobacterium Tuberculosis and Drug Resistance** | | | | | |
| --- | --- | --- | --- | --- | --- |
| 1. In your daily clinical work, you recommend drug resistance gene testing for patients with HIV co-infected with tuberculosis. | a. always | b. often | c. sometimes | d. rarely | e. never |
| 2. In your daily clinical work, you educate tuberculosis patients about methods and benefits of drug-resistant tuberculosis testing. | a. always | b. often | c. sometimes | d. rarely | e. never |
| 3. In daily life, when encountering a suspected tuberculosis patient, you recommend timely consultation at a specialized hospital. | a. always | b. often | c. sometimes | d. rarely | e. never |
| 4. In your daily clinical work, when a suspected HIV and tuberculosis co-infected patient is reluctant to undergo testing, you still fulfill your duty to inform. | a. always | b. often | c. sometimes | d. rarely | e. never |
| 5. In your daily clinical work, you proactively study knowledge and treatment guidelines related to HIV co-infection with Mycobacterium tuberculosis and drug resistance. | a. always | b. often | c. sometimes | d. rarely | e. never |
| 6. When receiving patients, you routinely inquire about their history of HIV infection and tuberculosis exposure. | a. always | b. often | c. sometimes | d. rarely | e. never |
| 7. You handle samples from patients with HIV co-infection and drug-resistant Mycobacterium tuberculosis according to standard protective measures. | a. always | b. often | c. sometimes | d. rarely | e. never |
| 8. You monitor patients’ drug resistance test results and adjust treatment accordingly. | a. always | b. often | c. sometimes | d. rarely | e. never |
