# Supplementary material for: Knowledge, attitudes, and practices of healthcare providers in Beijing regarding human immunodeficiency virus and tuberculosis co-infection: A multicenter cross-sectional study
Source: PLoS One. 2026 Feb 23;21(2):e0341132. doi: 10.1371/journal.pone.0341132 (PMC12928499; doi:10.1371/journal.pone.0341132)
Supplement: S2 Appendix — Supplementary table 2. Estimated total effect coefficient. Supplementary table 3. Distribution of knowledge dimension responses. Supplementary table 4. Distribution of attitude dimension responses. Supplementary table 5. Distribution of practice dimension responses. Supplementary table 6. SEM fit indicators. (DOCX) [file pone.0341132.s002.docx]

**Appendix 2 Supplementary Tables**

**Supplementary Table 1. Cronbach’s alpha coefficients for individual KAP items.**

|  | Scale Mean if Item Deleted | Scale Variance if Item Deleted | Corrected Item-Total Correlation | Cronbach's Alpha if Item Deleted |
| --- | --- | --- | --- | --- |
| K1 | 91.53 | 283.253 | 0.586 | 0.952 |
| K2 | 91.55 | 282.255 | 0.615 | 0.952 |
| K3 | 91.53 | 282.430 | 0.605 | 0.952 |
| K4 | 91.55 | 281.869 | 0.649 | 0.951 |
| K5 | 91.46 | 282.228 | 0.652 | 0.951 |
| K6 | 91.44 | 283.208 | 0.619 | 0.952 |
| K7 | 91.69 | 280.876 | 0.645 | 0.951 |
| K8 | 91.56 | 281.963 | 0.610 | 0.952 |
| K9 | 91.73 | 279.919 | 0.665 | 0.951 |
| K10 | 91.78 | 280.002 | 0.615 | 0.952 |
| K11 | 91.82 | 279.509 | 0.660 | 0.951 |
| K12 | 91.77 | 279.357 | 0.711 | 0.951 |
| K13 | 91.86 | 278.418 | 0.710 | 0.951 |
| K14 | 91.90 | 279.112 | 0.688 | 0.951 |
| K15 | 91.88 | 278.889 | 0.703 | 0.951 |
| A1 | 88.42 | 282.429 | 0.599 | 0.952 |
| A2 | 88.42 | 282.240 | 0.622 | 0.952 |
| A3 | 88.44 | 282.420 | 0.626 | 0.952 |
| A4 | 88.36 | 283.420 | 0.595 | 0.952 |
| A5 | 88.46 | 282.756 | 0.591 | 0.952 |
| A6 | 88.67 | 279.238 | 0.620 | 0.951 |
| A7 | 88.71 | 277.504 | 0.663 | 0.951 |
| A8 | 88.48 | 281.498 | 0.639 | 0.951 |
| A9 | 88.43 | 282.218 | 0.627 | 0.952 |
| A10 | 89.00 | 282.894 | 0.369 | 0.954 |
| P1 | 89.48 | 265.615 | 0.641 | 0.952 |
| P2 | 89.38 | 264.126 | 0.719 | 0.951 |
| P3 | 88.59 | 277.101 | 0.532 | 0.952 |
| P4 | 88.62 | 276.198 | 0.583 | 0.952 |
| P5 | 89.15 | 269.011 | 0.707 | 0.951 |
| P6 | 89.17 | 268.670 | 0.641 | 0.952 |
| P7 | 88.99 | 269.386 | 0.607 | 0.952 |
| P8 | 89.11 | 265.940 | 0.673 | 0.951 |

**Supplementary table 2. Estimated total effect coefficient.**

| **Variable** | **Direction** | **Dimensons** | **Estimate** | **Standardized Estimate** | **S.E.** | **C.R.** | **P** |
| --- | --- | --- | --- | --- | --- | --- | --- |
| Attitude | <--- | Knowledge | 0.446 | 0.458 | 0.061 | 7.347 | <0.001 |
| Practice | <--- | Attitude | 0.627 | 0.272 | 0.114 | 5.510 | <0.001 |
| Practice | <--- | Knowledge | 1.102 | 0.491 | 0.116 | 9.470 | <0.001 |
| K1 | <--- | Knowledge | 1.000 | 0.668 |  |  |  |
| K2 | <--- | Knowledge | 1.090 | 0.706 | 0.071 | 15.446 | <0.001 |
| K3 | <--- | Knowledge | 1.052 | 0.698 | 0.069 | 15.269 | <0.001 |
| K4 | <--- | Knowledge | 1.076 | 0.713 | 0.069 | 15.576 | <0.001 |
| K5 | <--- | Knowledge | 1.025 | 0.700 | 0.067 | 15.315 | <0.001 |
| K6 | <--- | Knowledge | 0.980 | 0.688 | 0.065 | 15.080 | <0.001 |
| K7 | <--- | Knowledge | 1.262 | 0.776 | 0.075 | 16.772 | <0.001 |
| K8 | <--- | Knowledge | 1.121 | 0.705 | 0.073 | 15.427 | <0.001 |
| K9 | <--- | Knowledge | 1.360 | 0.807 | 0.078 | 17.357 | <0.001 |
| K10 | <--- | Knowledge | 1.415 | 0.785 | 0.084 | 16.940 | <0.001 |
| K11 | <--- | Knowledge | 1.456 | 0.837 | 0.081 | 17.904 | <0.001 |
| K12 | <--- | Knowledge | 1.403 | 0.857 | 0.077 | 18.259 | <0.001 |
| K13 | <--- | Knowledge | 1.466 | 0.845 | 0.081 | 18.042 | <0.001 |
| K14 | <--- | Knowledge | 1.359 | 0.805 | 0.079 | 17.297 | <0.001 |
| K15 | <--- | Knowledge | 1.401 | 0.823 | 0.079 | 17.636 | <0.001 |
| A10 | <--- | Attitude | 1.000 | 0.415 |  |  |  |
| A9 | <--- | Attitude | 1.336 | 0.855 | 0.131 | 10.216 | <0.001 |
| A8 | <--- | Attitude | 1.363 | 0.843 | 0.134 | 10.178 | <0.001 |
| A7 | <--- | Attitude | 1.421 | 0.710 | 0.147 | 9.658 | <0.001 |
| A6 | <--- | Attitude | 1.395 | 0.724 | 0.143 | 9.723 | <0.001 |
| A5 | <--- | Attitude | 1.338 | 0.844 | 0.131 | 10.180 | <0.001 |
| A4 | <--- | Attitude | 1.349 | 0.903 | 0.130 | 10.357 | <0.001 |
| A3 | <--- | Attitude | 1.378 | 0.895 | 0.133 | 10.335 | <0.001 |
| A2 | <--- | Attitude | 1.391 | 0.885 | 0.135 | 10.307 | <0.001 |
| A1 | <--- | Attitude | 1.305 | 0.812 | 0.130 | 10.075 | <0.001 |
| P1 | <--- | Practice | 1.000 | 0.676 |  |  |  |
| P2 | <--- | Practice | 1.058 | 0.755 | 0.045 | 23.528 | <0.001 |
| P3 | <--- | Practice | 0.640 | 0.590 | 0.050 | 12.887 | <0.001 |
| P4 | <--- | Practice | 0.701 | 0.669 | 0.048 | 14.473 | <0.001 |
| P5 | <--- | Practice | 0.963 | 0.802 | 0.057 | 16.985 | <0.001 |
| P6 | <--- | Practice | 1.071 | 0.805 | 0.063 | 17.036 | <0.001 |
| P7 | <--- | Practice | 1.077 | 0.793 | 0.064 | 16.809 | <0.001 |
| P8 | <--- | Practice | 1.195 | 0.853 | 0.067 | 17.877 | <0.001 |

**Supplementary table 3. Distribution of knowledge dimension responses.**

| **Items** | **N (%)** | | |
| --- | --- | --- | --- |
|  | **Very familiar** | **Heard of it** | **Not clear** |
| **1. After being infected with Mycobacterium tuberculosis, the human body may develop either latent tuberculosis infection or active tuberculosis.** | 249 (44.07) | 278 (49.2) | 38 (6.73) |
| **2. HIV infection is an independent risk factor for developing tuberculosis, and people living with HIV have a significantly higher risk of latent tuberculosis infection progressing to active TB compared to those without HIV.** | 246 (43.54) | 273 (48.32) | 46 (8.14) |
| **3. Tuberculosis is one of the most common opportunistic infections among people living with HIV. It is a major factor in disease progression and a leading cause of death in AIDS patients.** | 257 (45.49) | 263 (46.55) | 45 (7.96) |
| **4. With the widespread use of anti-tuberculosis drugs, the emergence and spread of drug-resistant tuberculosis has become a global issue.** | 242 (42.83) | 282 (49.91) | 41 (7.26) |
| **5. Prompt identification of Mycobacterium tuberculosis infection is key to early clinical diagnosis and treatment.** | 279 (49.38) | 256 (45.31) | 30 (5.31) |
| **6. The gold standard for clinical diagnosis of Mycobacterium tuberculosis is the isolation of the organism from secretions or fluids (e.g., sputum, bronchoalveolar lavage fluid, or pleural effusion) or from tissues (e.g., pleural biopsy or lung biopsy), or a positive nucleic acid identification.** | 287 (50.8) | 254 (44.96) | 24 (4.25) |
| **7. The diagnosis of HIV/MTB co-infection is relatively more difficult, with atypical clinical manifestations. The presence of multiple other opportunistic infections complicates the condition, and extrapulmonary tuberculosis is relatively more common.** | 195 (34.51) | 294 (52.04) | 76 (13.45) |
| **8. Tuberculin skin tests and interferon-gamma release assays are specifically used for the diagnosis of tuberculosis infection.** | 246 (43.54) | 265 (46.9) | 54 (9.56) |
| **9. Molecular testing, such as gene probes and sequencing, can be used to detect Mycobacterium tuberculosis DNA and common mutations associated with drug resistance.** | 188 (33.27) | 285 (50.44) | 92 (16.28) |
| **10. Tuberculosis infection may occur in HIV-positive individuals regardless of their CD4+ T lymphocyte count.** | 191 (33.81) | 254 (44.96) | 120 (21.24) |
| **11. High-throughput sequencing is widely used in clinical practice for the diagnosis of tuberculosis infection and the detection of drug resistance.** | 166 (29.38) | 280 (49.56) | 119 (21.06) |
| **12. The treatment regimen for tuberculosis in people living with HIV is the same as for those with tuberculosis alone, but drug interactions between anti-TB and antiretroviral medications must be considered.** | 169 (29.91) | 305 (53.98) | 91 (16.11) |
| **13. For all newly diagnosed TB patients, routine drug susceptibility testing for first-line anti-tuberculosis drugs is recommended. For patients who remain culture-positive after four months of treatment or who become culture-positive again after initial conversion, repeated first-line drug susceptibility testing is recommended.** | 153 (27.08) | 284 (50.27) | 128 (22.65) |
| **14. Treatment failure in drug-susceptible tuberculosis refers to cases where the patient remains culture-positive after four months of anti-TB treatment (five months according to China, Europe, and WHO definitions).** | 138 (24.42) | 292 (51.68) | 135 (23.89) |
| **15. The treatment of multidrug-resistant TB (MDR-TB) and extensively drug-resistant TB (XDR-TB) should be individualized, taking into account the drug resistance pattern of MTB, the availability of anti-TB drugs, disease severity, and co-infections.** | 143 (25.31) | 295 (52.21) | 127 (22.48) |

**Supplementary table 4. Distribution of attitude dimension responses.**

| **Items** | **N (%)** | | | | |
| --- | --- | --- | --- | --- | --- |
|  | **Strongly agree** | **Agree** | **Neutral** | **Disagree** | **Strongly disagree** |
| **1. You believe that HIV co-infection with Mycobacterium tuberculosis and drug resistance poses a significant threat to public health.** | 313 (55.4) | 211 (37.35) | 40 (7.08) | 1 (0.18) |  |
| **2. You believe it is important to be well-versed in knowledge related to HIV co-infection with Mycobacterium tuberculosis and drug resistance.** | 310 (54.87) | 224 (39.65) | 29 (5.13) | 1 (0.18) | 1 (0.18) |
| **3. You believe that HIV co-infection with Mycobacterium tuberculosis and drug resistance is a complex condition.** | 298 (52.74) | 235 (41.59) | 31 (5.49) | 1 (0.18) |  |
| **4. You believe it is important to implement protocols to prevent the transmission of HIV co-infection with Mycobacterium tuberculosis and drug resistance.** | 332 (58.76) | 207 (36.64) | 25 (4.42) | 1 (0.18) |  |
| **5. You believe that the diagnosis of HIV co-infection with Mycobacterium tuberculosis and drug resistance should strictly follow guidelines.** | 290 (51.33) | 236 (41.77) | 38 (6.73) | 1 (0.18) |  |
| **6. You are very interested in new diagnostic technologies for HIV co-infection with Mycobacterium tuberculosis and drug resistance.** | 237 (41.95) | 229 (40.53) | 92 (16.28) | 7 (1.24) |  |
| **7. You are very interested in updates to treatment regimens for HIV co-infection with Mycobacterium tuberculosis and drug resistance.** | 232 (41.06) | 221 (39.12) | 102 (18.05) | 10 (1.77) |  |
| **8. You believe that managing patients with HIV co-infection and drug-resistant Mycobacterium tuberculosis is a challenging but meaningful task.** | 279 (49.38) | 251 (44.42) | 31 (5.49) | 3 (0.53) | 1 (0.18) |
| **9. You believe that increasing awareness of HIV co-infection with Mycobacterium tuberculosis and drug resistance can reduce the risk of transmission.** | 300 (53.1) | 234 (41.42) | 28 (4.96) | 3 (0.53) |  |
| **10. You believe that the training provided by hospitals on HIV co-infection with Mycobacterium tuberculosis and drug resistance is insufficient.** | 176 (31.15) | 207 (36.64) | 141 (24.96) | 34 (6.02) | 7 (1.24) |

**Supplementary table 5. Distribution of practice dimension responses.**

| **Items** | **N (%)** | | | | |
| --- | --- | --- | --- | --- | --- |
|  | **Always** | **Often** | **Sometimes** | **Rarely** | **Never** |
| **1. In your daily clinical work, you recommend drug resistance gene testing for patients with HIV co-infected with tuberculosis.** | 155 (27.43) | 152 (26.9) | 101 (17.88) | 91 (16.11) | 66 (11.68) |
| **2. In your daily clinical work, you educate tuberculosis patients about methods and benefits of drug-resistant tuberculosis testing.** | 166 (29.38) | 147 (26.02) | 111 (19.65) | 100 (17.7) | 41 (7.26) |
| **3. In daily life, when encountering a suspected tuberculosis patient, you recommend timely consultation at a specialized hospital.** | 326 (57.7) | 146 (25.84) | 50 (8.85) | 31 (5.49) | 12 (2.12) |
| **4. In your daily clinical work, when a suspected HIV and tuberculosis co-infected patient is reluctant to undergo testing, you still fulfill your duty to inform.** | 303 (53.63) | 169 (29.91) | 58 (10.27) | 23 (4.07) | 12 (2.12) |
| **5. In your daily clinical work, you proactively study knowledge and treatment guidelines related to HIV co-infection with Mycobacterium tuberculosis and drug resistance.** | 181 (32.04) | 158 (27.96) | 149 (26.37) | 63 (11.15) | 14 (2.48) |
| **6. When receiving patients, you routinely inquire about their history of HIV infection and tuberculosis exposure.** | 198 (35.04) | 155 (27.43) | 101 (17.88) | 86 (15.22) | 25 (4.42) |
| **7. You handle samples from patients with HIV co-infection and drug-resistant Mycobacterium tuberculosis according to standard protective measures.** | 250 (44.25) | 146 (25.84) | 67 (11.86) | 73 (12.92) | 29 (5.13) |
| **8. You monitor patients’ drug resistance test results and adjust treatment accordingly.** | 225 (39.82) | 148 (26.19) | 80 (14.16) | 74 (13.1) | 38 (6.73) |

**Supplementary table 6. SEM fit indicators.**

| **Model fit indicators** | **Ref.** | **Measured results** |
| --- | --- | --- |
| **CMIN/DF** | 1-3 excellent，3-5 good | 4.460 |
| **RMSEA** | <0.08 good | 0.078 |
| **IFI** | >0.8 good | 0.900 |
| **TLI** | >0.8 good | 0.891 |
| **CFI** | >0.8 good | 0.900 |
